# Trustworthy Augmented Intelligence in Health Care

**DOI:** 10.1007/s10916-021-01790-z

**Published:** 2022-01-12

**Authors:** Elliott Crigger, Karen Reinbold, Chelsea Hanson, Audiey Kao, Kathleen Blake, Mira Irons

**Affiliations:** American Medical Association, 330 North Wabash, Chicago, Il 60611 US

**Keywords:** Accountability, Augmented intelligence/artificial intelligence, Equity/access to care, Ethics, Health care innovation, Standards

## Abstract

Augmented Intelligence (AI) systems have the power to transform health care and bring us closer to the quadruple aim: enhancing patient experience, improving population health, reducing costs, and improving the work life of health care providers. Earning physicians' trust is critical for accelerating adoption of AI into patient care. As technology evolves, the medical community will need to develop standards for these innovative technologies and re-visit current regulatory systems that physicians and patients rely on to ensure that health care AI is responsible, evidence-based, free from bias, and designed and deployed to promote equity. To develop actionable guidance for trustworthy AI in health care, the AMA reviewed literature on the challenges health care AI poses and reflected on existing guidance as a starting point for addressing those challenges (including models for regulating the introduction of innovative technologies into clinical care).

## Introduction

Augmented Intelligence (AI) [1] systems have the power to transform health care by harnessing the promise of artificial intelligence to support clinicians and patients and bringing us closer to achieving the quadruple aim: enhancing patient experience, improving population health, reducing costs, and improving the work life of health care professionals [2]. Earning physicians’ trust is critical for accelerating adoption of AI into patient care. As technology evolves, the medical community will need to develop standards for evaluating, integrating, using and monitoring these innovative technologies. The regulatory systems and operational practices that have been the bedrock upon which physician and patient confidence in medical technology depend are now charged with ensuring that health care AI is evidence-based, free from bias, and promotes equity. As a leading voice in medical ethics and health policy, representing some 270,000 physicians and over 120 national medical specialty and other societies, the American Medical Association (AMA) is uniquely positioned to guide physicians, patients, and the broader health care community in the development and use of trustworthy AI.

## Defining trustworthy

Trustworthy means dependable and worthy of confidence [3]. In health care, this requires systematically building an evidence-base using rigorous, standardized processes for design, validation, implementation, and monitoring grounded in ethics and equity. The dangers of adopting AI without these guardrails were made abundantly clear in the recent example of an algorithm that used historical health care spending as a proxy for illness severity to predict an individual’s future health needs and establish their eligibility for additional services. This method excluded many Black patients from disease management programs, effectively expanding long-standing racial inequities in access to care [4].

To develop actionable guidance for trustworthy AI in health care, the AMA reviewed literature on the challenges health care AI poses and examined existing guidance as its starting point for addressing those challenges (including models for regulating the introduction of innovative technologies into clinical care). The literature and guidance confirm that AI must promote the ethical values of the medical profession, uphold exacting standards of scientific inquiry and evidence, and advance equity in health care.

## Ethics, evidence, and equity in health care

To merit the trust of patients and physicians, AI in health care must focus on matters of ethics, evidence, and equity.

### Ethics

Ethical AI must uphold the fundamental values of medicine as a profession and as a moral activity grounded in relationships between “someone who is ill, on the one hand, and someone who professes to heal on the other” [5]. While incorporating new technologies is expected in health care, AI-enabled technologies possess characteristics that set them apart from other innovations in ways that can impinge on a therapeutic patient-physician relationship. Notably, AI algorithms are trained on datasets of varying quality and completeness and are implemented across multiple environments and thus carry the risk of driving inequities in outcomes across patient populations. Further, the most powerful, and useful, AI systems are adaptive, able to learn and evolve over time outside of human observation and independent of human control [6], while accountability is diffused among the multiple stakeholders who are involved in design, development, deployment, and oversight and who have differing forms of expertise, understandings of professionalism, and goals [7].

Despite these new challenges, existing frameworks lay a foundation for the ethical design and deployment of AI in health care and can help guide our understanding of the current state of AI principles.

For example, guidance in the AMA *Code of Medical Ethics* on ethically sound innovation in medical practice (Opinion 1.2.11) provides that any innovation intended to directly affect patient care be scientifically well grounded and developed in coordination with individuals who have appropriate clinical expertise; that the risks an innovation poses to individual patients should be minimized, and the likelihood that the innovation can be applied to and benefit populations of patients be maximized [8]. Opinion 1.2.11 further requires that meaningful oversight be ensured—not only in the development of an innovation, but in how it is integrated into the delivery of care.

The *Code* further addresses issues in the deployment of AI in Opinion 11.2.1, “Professionalism in Health Care Systems,” which emphasizes the ethical need to continuously monitor tools and practices deployed to organize the delivery of care to identify and address adverse consequences and to disseminate outcomes, positive and negative [9]. Opinion 11.2.1 explicitly requires that mechanisms designed to influence the provision of care not disadvantage identifiable populations of patients or exacerbate existing health care disparities and that they be implemented in conjunction with the resources and infrastructure needed to support high value care and professionalism. Institutional oversight should be sensitive to the possibility that even well-intended use of well-designed tools can lead to unintended consequences outside the clinical realm—in the specific context of AI, for example, when the use of clinical prediction models identifies individuals at risk for medical conditions that are stigmatizing or associated with discrimination against individuals or communities.

*Ethics Guidelines for Trustworthy AI* published by the European Commission’s High-Level Expert Group on Artificial Intelligence in 2019 highlights the essential role of trust in the development and adoption of AI and proposes a framework for achieving it [10].

The report states that trustworthy AI should be lawful, ethical, and robust. It should be based on human-centered design and adhere to ethical principles throughout its life cycle: respect for human autonomy, prevention of harm, fairness, and explicability. This report cautions that AI systems may pose risks that can be difficult to predict or observe and raises awareness about potential impacts on vulnerable populations. The report maintains that trustworthy AI requires a holistic approach involving all parties and processes, both technical and societal.

The European Parliamentary Research Service recently published a study, *Artificial Intelligence: From Ethics to Policy* that conceptualizes AI as a “real-world experiment” full of both risks and potential benefits [11]. In this framing, AI systems must meet the conditions for ethically responsible research: they must protect humans, assess predicted benefits, and appropriately balance these benefits against the risks AI systems pose to individuals and society. As in the *Ethics Guidelines for Trustworthy AI*, AI is viewed as a socio-technical system. It should be evaluated within the context of the society in which it is created. Recognizing that technology not only reinforces the way the world works today but can dictate the way it will work in the future, the report stresses the importance of incorporating ethics as an explicit consideration throughout the design, development, and implementation of AI.

### Evidence

To date, the evidence base for health care AI has focused primarily on the validation of AI algorithms, and a review of the literature reveals a lack of consistency in terminology and approach [12]. To strengthen the evidence base and earn the trust of patients and physicians, AI must systematically show that it meets the highest standards for scientific inquiry in design and development and must provide clinically relevant evidence of safety and effectiveness.

Existing frameworks for designing, conducting, and evaluating clinical research, such as the development process for drugs and devices approved by the U.S. Food & Drug Administration [13–15] offer a model on which to ground a standardized approach to meet this responsibility. At a minimum, an AI system intended for use in clinical care must demonstrate, first, that it is the product of a design protocol that addresses clearly defined, clinically relevant questions and objectives, and a well-documented, scientifically rigorous, and consistent validation process that demonstrates safety and efficacy. Then, that the AI system has been reviewed by a diverse team of well-qualified subject matter experts, and transparently reported in keeping with standards for scientific publication as discussed below.

Given the unique nature of AI, we must be prepared to revisit and refine these core requirements as technology evolves. A review of the literature shows that there are multiple approaches to evaluating the quality and level of evidence needed in health care applications. GRADE (Grading of Recommendations, Assessment, Development and Evaluation) is a method of rating the quality of evidence and the strength of clinical practice recommendations [16]. The International Medical Device Regulators Forum (IMDRF) has developed a risk categorization framework for Software as a Medical Device (SaMD) that assigns an impact level (category I – IV) to SaMDs based on two major factors: the significance of the information the tool provides to the health care decision and the state of the health care situation or condition [17, 18]. These types of evidence and risk frameworks can inform the levels of validation and evidence required for AI systems and address many of the ethical considerations that have been raised in the literature, including socio-technical environment considerations. The IMDRF framework also stresses the importance of post-market surveillance through a continuous learning process driven by real-world evidence. Recognizing that the use of AI in health care can range from administrative tasks to algorithms that inform diagnosis or treatment, it is critical that the level of evidence required be proportional to the degree of risk an AI system may pose to patients.

#### Bias

Given its centrality to concerns about AI in health care it is appropriate to draw attention briefly to the potential for bias in the design, operation, or deployment of adaptive systems in clinical settings [19, 20]. Algorithms trained on electronic health records (EHRs), as most currently are, risk building into the model itself whatever flaws exist in the record [21]: EHRs capture information only from individuals who have access to care and whose data are captured electronically; data are not uniformly structured across EHRs; and the majority of data in EHRs reflect information captured “downstream” of human judgments, with the risk that the model will replicate human cognitive errors [21, 22]. Moreover, well-intended efforts to correct for possible bias in training data can have unintended consequences, as is the case when “race-corrected” algorithms direct resources away from patients from minoritized populations rather than provide equitable personalized care [23].

Efforts to build fair adaptive models must meet challenges of mathematically defining “fairness” in the first place, [24, 25] and of determining just what trade-offs between fairness and model performance are acceptable [25]. Beyond these challenges, even algorithms that are, hypothetically, fair out of the box may become biased over time when they are deployed in contexts different from those in which they were created, or when they “learn from pervasive, ongoing, and uncorrected biases in the broader health care system” [19]. Models may be followed uncritically, or be implemented only in certain settings such that they disproportionately benefit individuals “who are already experiencing privilege of one sort or another.” Finally, they may preferentially select or encourage outcomes that “do not reflect the interests of individual patients or the community” [19].

### Equity

The AMA’s vision for health equity is a nation where all people live in thriving communities where resources work well, systems are equitable and create no harm, everyone has the power to achieve optimal health, and all physicians are equipped with the consciousness, tools, and resources to confront inequities as well as embed and advance equity within and across all aspects of the health care system. While great opportunity exists for technological innovations to advance health equity, current models of resource allocation, evidence development, solution design, and market selection fail to incorporate an equity lens – risking the automation, scaling, and exacerbation of health disparities rooted in historical and contemporary racial and social injustices.

Equity issues arise when the data set used to train an algorithm excludes or underrepresents historically marginalized and minoritized patient populations, failing to account for significant differences in experience or outcomes associated with patient identity. The design of the algorithm itself might exacerbate inequities if proxies or assumptions are based in historical discrimination and injustices, as illustrated by the disease management algorithm cited above.

So too, while algorithms are often exalted as more objective than humans, they are developed by humans who are inherently biased [26]. Solution design and development in venture-backed startups, large technology companies, and academic medical centers often lack representation of marginalized communities – with Black, Latinx, LGBTQ + , people with disabilities, and other populations excluded from resourced innovation teams and in user testing efforts.

The 2018 report on AI in health care by AMA’s Board of Trustees recognized that one of the most significant implications for end users of AI systems is that these systems can, invisibly and unintentionally, “reproduce and normalize” the biases of their training data sets [1]. Sociologist and Princeton University Professor, Ruha Benjamin, PhD in her book, *Race After Technology* presents several powerful examples of how “coded inequities…hide, speed up, and even deepen discrimination, while appearing to be neutral or benevolent when compared to the racism of a previous era.” She also discusses lack of intentionality as an inadequate excuse for perpetuation of biases and discrimination [27].

The implications for those developing and evaluating health care AI solutions are that an equity lens must be applied intentionally from the very beginning – in populating the design and testing team, the framing of the problem to be solved, the training data set selected, and the design and evaluation of the algorithm itself. This challenge to developers and evaluators aligns with the European Commission’s *Statement on Artificial Intelligence, Robotics and ‘Autonomous’ Systems* that “Discriminatory biases in data sets used to train and run AI systems should be prevented or detected, reported and neutralized at the earliest stage possible”﻿ [28]. It is also critical that we recognize AI as a downstream lever connected to larger upstream issues of inequity in our health system. Even if AI solutions are designed with a more intentional equity lens, we must understand that their deployment is within a system that distributes resources and allocates opportunities for optimal health and wellbeing to some communities at the expense of others. As powerful advocates for patients, physicians have an opportunity to look upstream and ask not just about the design of the algorithm itself but what it will mean for the health and care of patients in the environment within which it is implemented.

## Current state of AI guidelines and regulations

A recent publication from Harvard University’s Berkman Klein Center for Internet & Society is a survey of AI principles documents that have been published around the globe in recent years [29], including the OECD Principles on Artificial Intelligence that the United States and 41 other nations adopted in 2019 [30]. Table 1 summarizes common themes in these guidelines and regulations.

**Table 1:** Common themes from AI guidelines and regulations

Privacy﻿	Data subjects should have come degree of influence over how and why information about them is used
Accountability	AI systems should be subject to oversight during development and deployment; right remedies should be provided if harm occurs
Safety and Security	AI systems must be reliable and perform as intended’ systems must be appropriately protected against external threats
Transparency and explainability	It must be clear when AI systems are being used and for what task’ justifications for decision outputs should be intelligible
Fairness and non-discrimination	Steps should be taken to prevent and mitigate against discrimination risks in the design, development, and application of AI systems
Human control of technology	Important decisions are still subject to human control
Professional responsibility	Individuals and teams involved in the development and deployment of AI systems take responsibility for the performance and effects of those systems
Promotion of human values	The ends to which AI systems are devoted and how they are implemented and should correspond with core social norms

The report offers a comprehensive picture of the key principles that underlie each theme and can serve as a valuable resource for the development of standards that apply to AI systems intended for use by physicians, patients, and health systems.

The European Parliament Research Service study, *Artificial Intelligence: From Ethics to Policy*, proposes concrete steps that can be taken to address ethics concerns [11]. These include requiring developers to hold a data hygiene certificate at the organization-level that ensures data quality without requiring the disclosure of proprietary algorithms or data sets; requiring institutions deploying AI to conduct an ethical technology assessment prior to deployment to ensure that ethical issues have been considered; and completing an accountability report post-deployment to document how they have mitigated or corrected the concerns raised in the assessment.

The European Commission’s *Ethics Guidelines for Trustworthy AI* proposes seven requirements that AI systems should meet and provides a list of assessments that can help organizations operationalize these requirements [10].

In the context of health care, the guidance entitled *Software as a Medical Device (SaMD): Clinical Evaluation* issued by the International Medical Device Regulators Forum (IMDRF), in which the FDA Center for Devices and Radiological Health is an active participant, is particularly valuable [17]. Clinical evaluation includes the gathering and assessment of scientific validity, analytical validity, and clinical performance (real-world patient data). This guidance provides examples of relevant clinical evaluation methods and processes that can be used for SaMD. It also describes the level of evidence that should be required for different patient risk categories and identifies circumstances when independent review is important. For example, it suggests that SaMD categorized as negligible risk may only require scientific and analytical validity whereas SaMD that is categorized as high-risk would require clinical performance data in addition to scientific and analytical validity. Independent review recommendations are similarly tiered based on risk categorization.

Standard-setting and regulatory bodies will need to balance competing demands for protecting patient safety and advancing innovation because unsafe innovation could lead, fairly or unfairly, to lack of trust in all AI products and loss of the benefit to patients of trustworthy AI products. The FDA’s Digital Health Innovation Action Plan [31] outlines steps the regulatory agency is taking towards achieving this balance. FDA is modernizing its policies [32], increasing its digital health staff, and has launched a Digital Health Software Precertification Pilot Program or “Pre-Cert” designed to test a more efficient, streamlined pathway with a shortened approval timeline for entities who demonstrate “organizational excellence.” To support these efforts, the FDA established a Digital Health Center of Excellence where developers, regulators and the public can access digital health resources and expertise [33]. The Agency has leveraged IMDFR guidance to propose a new total product lifecycle or TPLC regulatory framework that would better position the FDA to regulate adaptive AI and Machine Learning (ML) technologies [34]. It is worth noting that the FDA’s regulatory authority only applies to AI and ML tools that meet the definition of a medical device [35].

A recent systematic review of studies evaluating the performance of diagnostic deep learning algorithms for medical imaging points to the need for greater transparency and standardization in reporting [36]. Most studies reviewed were based on non-randomized clinical trials that were at elevated risk of bias and did not follow reporting standards, making it challenging to evaluate the conclusions made. Several initiatives are underway to address this. The Consolidated Standards for Reporting Trials (CONSORT) and Standard Protocol Items: Recommendations for Interventional Trials (SPIRIT) provide minimum reporting guidelines for randomized trials and trial protocols. A working group recently published CONSORT-AI and SPIRIT-AI guidelines that extend the original statements to address challenges and issues specific to AI [12]. These international, consensus-based guidelines are based on the Enhancing Quality and Transparency in Health Research’s (EQUATOR) Network methodology for developing guidelines. Acceptance of these standards hinges on adoption by scientific journals, many of which have required authors to comply with CONSORT and SPIRIT standards in the past. Other ongoing efforts include a machine learning-focused version of the Transparent Reporting of a Multivariable Prediction Model for Individual Prognosis or Diagnosis statement (TRIPOD-ML) [37] and Minimum Information for Medical AI Reporting (MINIMAR) [38]. These efforts to set minimum requirements and standards for reporting are a major step toward promoting transparency and reproducibility. The recent publication of a new American National Standards Institute (ANSI)-accredited standard from the Consumer Technology Association on AI in health care further supports transparency by providing a framework with common definitions that stakeholders can use to improve understanding [39].

The emerging consensus around core issues suggests that responsible use of AI in medicine entails commitment to designing and deploying AI systems that address clinically meaningful goals, upholding the profession-defining values of medicine, promoting health equity, supporting meaningful oversight and monitoring of system performance, and establishing clear expectations for accountability and mechanisms for holding stakeholders accountable. Education and training efforts are also needed to increase the number and diversity of physicians with AI knowledge and expertise.

Artificial intelligence is not synonymous with augmented intelligence. Artificial intelligence “constitutes a host of computational methods that produce systems that perform tasks normally requiring human intelligence. These computational methods include, but are not limited to, machine image recognition, natural language processing, and machine learning. However, in health care a more appropriate term is ‘augmented intelligence,’ reflecting the enhanced capabilities of human clinical decision making when coupled with these computational methods and systems” [1]. “Artificial intelligence” is a tool that produces an output;” augmented intelligence” combines human intelligence and machine-derived outputs to improve health.

As with many of the tools used in patient care, physicians often serve as trusted intermediaries and are expected to understand and communicate the benefits, risk, indications, appropriateness, and alternatives of use. This process of understanding and communicating is fulfilled at the individual patient level in the exam room and at the organizational level when new products are reviewed by institutional purchasing committees, analogous to existing pharmacy and therapeutics committees.. Health technology assessment organizations and health plans focus their analyses less on individuals and more on populations with a greater emphasis on economic and cost–benefit considerations than might be seen in the clinical realm. Due diligence is expected, and indeed required, of all who are empowered to make acquisition, implementation and coverage decisions; it is assumed, perhaps implicitly, by patients.

For practicing physicians, lifelong learning includes understanding for whom, when and how new technologies such as AI will improve health and health care. A clinician’s qualifications to practice in their specialty are verified by hospital credentialing committees, health plans, certification bodies, state licensing boards and others. Therefore, in order to serve their patients and to be qualified and credentialed to practice in an environment in which AI tools are used, physicians must understand enough, albeit not everything, about new tools, and devices in their practice. If the “box is too black,” such that an artificial intelligence product is not or cannot be explained, it will be difficult for physicians responsible for evaluating, selecting, and implementing such products to recommend use, even if that means foregoing the potential benefits to patient health that might otherwise be achieved.

## Translating principles into practice: Framework for building ai that physicians can trust

Clearly defining roles and responsibilities among those who develop clinical AI systems, health care organizations and leaders who deploy those in clinical settings, and physicians who integrate AI into care for individual patients is central to putting the ethics-evidence-equity framework into practice. In the first instance, stakeholders must jointly ensure that a diverse community of patients and physicians are engaged throughout the process, all parties align on best practices, oversight, and accountability, and physicians and the public are educated to be informed and empowered consumers of health care AI. Table 2 delineates further the cross-cutting responsibilities of developers, deployers, and end users in fulfilling commitments to ethics, evidence, and equity.

**Table 2:** Crosscutting responsibilities of developers, deployers, and end users in fulfilling commitments to ethics, evidence, and equity

Responsibility	Developer	Deployer	Physician
***Planning and development***			
Ensure the AI system addresses a meaningful clinical goal	☑		☑
Ensure the AI system works as intended	☑		☑
Explore and resolve legal implications of the AI system [a]^a^ prior to implementation and agree upon professional and/or governmental oversight for safe, effective, and fair use of and access to health care AI	☑	☑	☑
Develop a clear protocol to identify and correct for potential bias	☑	☑	☑
Ensure appropriate patient safeguards are in place for direct-to-consumer tools that lack physician oversight	☑		
***Implementation and monitoring***			
Make clinical decisions such as diagnosis and treatment			☑
Have the authority and ability to override the AI system			☑
Ensure meaningful oversight is in place for ongoing monitoring		☑	☑
Ensure the AI system continues to perform as intended through performance monitoring & maintenance	☑	☑	
Ensure ethical issues identified at the time of purchase and during use have been addressed.^b^		☑	
Ensure clear protocols exist for enforcement and accountability, including a clear protocol to ensure equitable implementation	☑	☑	☑

^a^Such as issues of liability or intellectual property

^b^Including but not limited to safeguarding patients’ and other individuals’ privacy interests and preserving the security and integrity of personal information; securing patient consent; and providing patients’ access to records

Successfully integrating AI into health care requires collaboration, and engaging stakeholders early to address these issues is critical.

Several efforts exist to support patient engagement in AI solution design, including but not limited to the Algorithmic Justice League https://www.ajl.org/, Data 4 Black Lives https://d4bl.org/about.html, #MoreThanCode https://morethancode.cc/about/, The Just Data Lab https://www.thejustdatalab.com/, and Auditing Algorithms https://auditingalgorithms.science/.)

To promote physician engagement, the AMA has developed the Physician Innovation Network. This online platform connects health care solution developers and physicians to ensure that physician input is integrated into health innovation solution design across the industry [40]. Engaging physicians at the early development stage can help ensure that AI systems are designed and implemented in a manner that upholds the ethical values of medicine and promotes the quadruple aim (Table 3).

**Table 3:** Trustworthy augmented intelligence in the context of the quadruple aim

Aim 1﻿. Enhancing patient experience Patient rights are respected, they are empowered to make an informed decision about the use of AI in their care, and research results improve their clinical outcomes, quality of life and satisfaction
Aim 2. Improving population health Health care AI addresses high-priority clinical needs and advances health equity by reducing disparities rooted in historical and contemporary injustice and discrimination, helping all patients inclusive of identity and socioeconomic factors
Aim 3. Reducing cost Oversight and regulatory structures account for the risk of harm and benefit posed by healthcare AI systems. Payment and coverage on following laws and regulations, providing appropriate levels of clinical validation and high-quality evidence, and advancing affordability and access
Aim 4. Improving the work life of health care providers Physicians are engaged in developing and implementing health care AI tools that augment their ability to provide high-quality clinically validated health care to patients and improve their well-being. Barriers to adoption such as lack of education on AI and liability and payment issues are resolved

Practicing physicians should use the following framework to evaluate whether an AI innovation meets these conditions: does it work, does it work for my patients, and does it improve health outcomes? The comments under each question supply guidance to address key issues found in the interviews (Appendix I). This framework can serve as a mental checklist for physicians and can help developers and deployers understand what is required to meet these expectations.

### Does it work?

The AI system meets expectations for ethics, evidence, and equity. It can be trusted as safe and effective.

The AI system was

- developed in response to a clearly defined clinical need identified by physicians and it addresses this need;
- designed, validated, and implemented with the physician’s perspective in mind.
- validated through a process commensurate with its risk [18].
  - It has been validated analytically and scientifically. An AI system that diagnoses or treats (i.e., is considerable risk) has been prospectively clinically validated in an appropriate care setting [4].
  - It has been tested for usability by participants who are demographically representative of end users.
  - The data and validation processes used to develop the AI system are known (i.e., publicly available).
  - It has received FDA approval or clearance (if applicable).

The developer

- has demonstrated that a predictive model predicts events early enough to meaningfully influence care decisions and outcomes,
- has an established commitment to data quality and security,
- has identified and addressed ethical considerations (e.g., an ethical technology assessment) [14],
- has robust data privacy and security processes in place for any patient data collected directly or from practice settings (i.e., for research or monitoring purposes),
- has identified and taken steps to address bias and avoided introducing or exacerbating health care disparities when testing or deploying the AI system, particularly among vulnerable populations,
- has ensured that the characteristics of the training dataset are known, and that the dataset reflects the diversity of the intended patient population, including demographic and geographic characteristics,
- has a transparent revalidation process in place for evaluating updates throughout the AI system’s lifecycle.

### Does it work for my patients?

The AI system has been shown to improve care for a patient population like mine, and I have the resources and infrastructure to implement it in an ethical and equitable manner.

- The AI system has been validated in a population and health care setting that reflects my practice.
- Continuous performance monitoring is in place in my practice to identify and communicate changes in performance to the developer
- It can be integrated smoothly into my current practice, will improve care, and will enhance my relationship with patients [5]
- The AI system has been beta tested in different populations prior to implementation to identify hidden bias.

### Does it improve health outcomes?

The AI system has been demonstrated to improve outcomes.

- Clinical performance and patient experience data demonstrate its positive impact on health outcomes, including quality of life measures, through qualitative and quantitative research methods.
- The AI system maximizes benefits and minimizes harm to patients, with particular attention to potential impacts on historically marginalized communities.
- The AI system improves patient well-being and experience, as defined by a diverse patient population.
- The AI system adds value to the physician–patient relationship, enabling patient-centered care.
- If the AI system only improves patient outcomes for specific populations, this limitation is transparent.
- Barriers to access are found and addressed to improve outcomes for all patients who can benefit.

All parties are responsible for ensuring that stakeholders are held accountable for meeting these expectations.

## Conclusion

While the number of AI systems used in health care has increased exponentially in recent years and numerous frameworks for ethical use and development of AI have been proposed, there is still no consensus on guiding principles for development and deployment of AI in health care. To harness the benefits that innovative technologies like AI can bring to health care, all stakeholders must work together to build the evidence, oversight, and infrastructure necessary to foster trust.

The guidance presented above provides a framework for development and use of AI through the lens of the patient-physician encounter. This framework promotes an evidence-based, ethical approach that advances health equity in support of the Quadruple Aim and reinforces the core values of medicine.

Physicians have an ethical responsibility to place patient welfare above their own self-interest or obligations to others, to use sound medical judgment on patients’ behalf, and to advocate for patients’ welfare. Innovations in health care should sustain this fundamental responsibility of fidelity to patients. Those who design and deploy new interventions or technologies, particularly interventions or technologies intended to directly interface with decisions about patient care, have a responsibility to ensure that their work serves these goals. The framework outlined here provides the profession’s perspective on the conditions necessary to create a trustworthy environment for adopting AI in health care with a primary focus on patient safety and outcomes of care.

## Appendix: AMA policies on augmented intelligence

### Augmented intelligence in health care H-480.940

As a leader in American medicine, our AMA has a unique opportunity to ensure that the evolution of augmented intelligence (AI) in medicine benefits patients, physicians, and the health care community.

To that end our AMA will seek to:

1. Leverage its ongoing engagement in digital health and other priority areas for improving patient outcomes and physicians’ professional satisfaction to help set priorities for health care AI.
2. Identify opportunities to integrate the perspective of practicing physicians into the development, design, validation, and implementation of health care AI.
3. Promote development of thoughtfully designed, high-quality, clinically validated health care AI that:

- is designed and evaluated in keeping with best practices in user-centered design, particularly for physicians and other members of the health care team.
- is transparent.
- conforms to leading standards for reproducibility.
- identifies and takes steps to address bias and avoids introducing or exacerbating health care disparities including when testing or deploying new AI tools on vulnerable populations; and
- safeguards patients’ and other individuals’ privacy interests and preserves the security and integrity of personal information.

4. Encourage education for patients, physicians, medical students, other health care professionals, and health administrators to promote greater understanding of the promise and limitations of health care AI.

5. Explore the legal implications of health care AI, such as issues of liability or intellectual property, and advocate for appropriate professional and governmental oversight for safe, effective, and equitable use of and access to health care AI.

## Augmented intelligence in health care H-480.939

Our AMA supports the use and payment of augmented intelligence (AI) systems that advance the quadruple aim. AI systems should enhance the patient experience of care and outcomes, improve population health, reduce overall costs for the health care system while increasing value, and support the professional satisfaction of physicians and the health care team. To that end our AMA will advocate that:

1. Oversight and regulation of health care AI systems must be based on risk of harm and benefit accounting for a host of factors, including but not limited to: intended and reasonably expected use(s); evidence of safety, efficacy, and equity including addressing bias; AI system methods; level of automation; transparency; and conditions of deployment.
2. Payment and coverage for all health care AI systems must be conditioned on complying with all appropriate federal and state laws and regulations, including, but not limited to those governing patient safety, efficacy, equity, truthful claims, privacy, and security as well as state medical practice and licensure laws.
3. Payment and coverage for health care AI systems intended for clinical care must be conditioned on (a) clinical validation; (b) alignment with clinical decision-making that is familiar to physicians; and (c) high-quality clinical evidence.
4. Payment and coverage for health care AI systems must (a) be informed by real world workflow and human-centered design principles; (b) enable physicians to prepare for and transition to new care delivery models; (c) support effective communication and engagement between patients, physicians, and the health care team; (d) seamlessly integrate clinical, administrative, and population health management functions into workflow; and (e) seek end-user feedback to support iterative product improvement.
5. Payment and coverage policies must advance affordability and access to AI systems that are designed for small physician practices and patients and not limited to large practices and institutions. Government-conferred exclusivities and intellectual property laws are meant to foster innovation, but constitute interventions into the free market, and therefore, should be appropriately balanced with the need for competition, access, and affordability.
6. Physicians should not be penalized if they do not use AI systems while regulatory oversight, standards, clinical validation, clinical usefulness, and standards of care are in flux. Furthermore, our AMA opposes:

- Policies by payers, hospitals, health systems, or governmental entities that mandate use of health care AI systems as a condition of licensure, participation, payment, or coverage.
- The imposition of costs associated with acquisition, implementation, and maintenance of healthcare AI systems on physicians without sufficient payment.

7. Liability and incentives should be aligned so that the individual(s) or entity(ies) best positioned to know the AI system risks and best positioned to avert or mitigate harm do so through design, development, validation, and implementation. Our AMA will further advocate:

- Where a mandated use of AI systems prevents mitigation of risk and harm, the individual or entity issuing the mandate must be assigned all applicable liability.
- Developers of autonomous AI systems with clinical applications (screening, diagnosis, treatment) are in the best position to manage issues of liability arising directly from system failure or misdiagnosis and must accept this liability with measures such as maintaining appropriate medical liability insurance and in their agreements with users.
- Health care AI systems that are subject to non-disclosure agreements concerning flaws, malfunctions, or patient harm (referred to as gag clauses) must not be covered or paid and the party initiating or enforcing the gag clause assumes liability for any harm.

8. Our AMA, national medical specialty societies, and state medical associations—

- Identify areas of medical practice where AI systems would advance the quadruple aim.
- Leverage existing expertise to ensure clinical validation and clinical assessment of clinical applications of AI systems by medical experts.
- Outline new professional roles and capacities required to aid and guide health care AI systems; and
- Develop practice guidelines for clinical applications of AI systems.

9. There should be federal and state interagency collaboration with participation of the physician community and other stakeholders to advance the broader infrastructural capabilities and requirements necessary for AI solutions in health care to be sufficiently inclusive to benefit all patients, physicians, and other health care stakeholders.
10. AI is designed to enhance human intelligence and the patient-physician relationship rather than replace it.

### Augmented intelligence in medical education H-295.857

Our AMA encourages:

1. accrediting and licensing bodies to study how AI should be most appropriately addressed in accrediting and licensing standards.
2. medical specialty societies and boards to consider production of specialty-specific educational modules related to AI.
3. research regarding the effectiveness of AI instruction in medical education on learning and clinical outcomes.
4. institutions and programs to be deliberative in the determination of when AI-assisted technologies should be taught, including consideration of established evidence-based treatments, and including consideration regarding what other curricula may need to be eliminated to accommodate new training modules.
5. stakeholders to provide educational materials to help learners guard against inadvertent dissemination of bias that may be inherent in AI systems.
6. the study of how differences in institutional access to AI may impact disparities in education for students at schools with fewer resources and less access to AI technologies.
7. enhanced training across the continuum of medical education regarding assessment, understanding, and application of data in the care of patients.
8. the study of how disparities in AI educational resources may impact health care disparities for patients in communities with fewer resources and less access to AI technologies.
9. institutional leaders and academic deans to proactively accelerate the inclusion of nonclinicians, such as data scientists and engineers, onto their faculty rosters to assist learners in their understanding and use of AI; and.
10. close collaboration with and oversight by practicing physicians in the development of AI applications.

## Declarations

### Disclaimer

Except as noted in text, the views expressed are those of the authors and do not represent adopted AMA policy.
